# Turnover of human fat cells and their lipid content

**DOI:** 10.1186/1751-0147-57-S1-K1

**Published:** 2015-09-25

**Authors:** Peter Arner

**Affiliations:** 1 Department of Medicine, Karolinska Institutet, Solna, Sweden

## Introduction

The turnover of fat cells and their lipid content is constantly ongoing in adult life. Fat cell turnover has importance for development of adipose tissue consisting of many small fat cells (hyperplasia) or few but large cells (hypertrophy). The latter is linked to metabolic disorders. Variations in lipids turnover in fat cells is also linked to metabolic disease.

## Objectives

We are interested in measuring the turnover of human fat cells and their lipid content and study their impact on hyperplasia/hypertrophy and its clinical consequences.

## Methods

Lipid and fat cell turnover is measured by incorporation of atmospheric 14C into triglycerides and DNA of human fat cells. Hyperplasia/hypertrophy is determined by measuring fat mass and fat cell size.

## Results

Decreased turnover of fat cells and their lipid contents is associated with insulin resistance, dyslipidemia and altered metabolic function of fat cells. Low fat cell turnover leads to adipose hypertrophy which is linked to insulin resistance in subcutaneous fat and to dyslipidemia in visceral fat. A transcription factor (EBF-1) regulates hyperplasia/hypertrophy in human adipose tissue and in animal models. Changes in adipose EBF-1 expression is linked to abnormal metabolic function of fat cells and to in vivo insulin resistance.

## Conclusions

Altered turnover of fat cells and their lipid content is important for common clinical disorders.

